# Inhibition of endoplasmic reticulum stress prevents high‐fat diet mediated atrial fibrosis and fibrillation

**DOI:** 10.1111/jcmm.15816

**Published:** 2020-11-01

**Authors:** Yan Zhang, Shuwen Yang, Jing Fu, Annan Liu, Deping Liu, Suyan Cao

**Affiliations:** ^1^ Department of General Practice/VIP Medical Service Beijing Hospital National Center of Gerontology Beijing China; ^2^ Institute of Geriatric Medicine Chinese Academy of Medical Sciences Beijing China; ^3^ Department of Cardiology Beijing Hospital National Center of Gerontology Beijing China

**Keywords:** atrial fibrillation, atrial fibrosis, endoplasmic reticulum stress, high‐fat diet

## Abstract

Obesity is a significant risk factor for atrial fibrillation (AF), which is the most common sustained arrhythmia with increased mortality and morbidity. High‐fat diet (HFD)‐induced obesity is associated with the activation of endoplasmic reticulum stress (ERS). However, the role of ERS in HFD‐induced AF remains elusive. Human atrium samples were examined for the ERS activation test. C57BL/6J mice were divided into four groups, including the control group, the HFD group, the 4‐phenylbutyric acid (4‐PBA) group, and the HFD + 4‐PBA group. At the age of 4 weeks, the HFD group and the HFD + 4‐PBA group were given HFD to construct the obesity model, while the other two groups were given a normal diet (ND). Transesophageal programmed electrical stimulation was conducted to evaluate the AF inducibility and duration. Atrial fibrosis and ERS activation were also investigated.We found that CHOP and GRP‐78 protein were significantly higher in overweight patients than the controls (both *P* < 0.05). AF inducibility and duration of the HFD group were significantly higher than the other groups (both *P* < 0.05), while there was no difference between those groups (*P* > 0.05). The mice of the HFD group had significantly higher collagen volume fraction (CVF%) than the other groups (*P* < 0.05). ERS marker protein of GRP78, p‐PERK, ATF6 and CHOP protein expression level was increased in the HFD group, which were significantly mitigated in the HFD + 4‐PBA group. In summary, HFD‐induced ERS activation facilitates atrial fibrosis and AF. The inhibition of ERS might alleviate atrial fibrosis and reduce the incidence of AF‐associated obesity.

## 1 INTRODUCTION

Despite the exponential growth of knowledge in the clinical treatment of atrial fibrillation (AF), the underlying mechanism remains not entirely elucidated, which Limits the clinical efficacy.^1^ Obesity, as a worldwide health problem with epidemic proportions, has been demonstrated to be an essential risk factor for AF.^2^ It is reported that the overweight population suffers higher incidence, prevalence, severity and progression of AF compared with their normal‐weight counterparts, while weight loss could significantly reverse atrial remodelling and decreases AF burden and AF recurrence following treatment.^3^ The strong links between obesity and AF may provide some clues for the pathophysiological mechanism for AF.

Oxidative stress and inflammation are now considered as the central mediator of atrial fibrillation in obesity and diabetes, which lays the foundation of atrial remodelling and facilitates AF.^4^ Endoplasmic reticulum stress (ERS) denotes a disturbance of the protein folding process in the endoplasmic reticulum, leading to the activation of unfolded protein response (UPR).^5^ Long‐lasting and high levels of ERS and UPR induces cells to commit to self‐destruction, which is associated with a series of human diseases, including diabetes, neurodegeneration, cancer and cardiovascular disease. As a key contributor, ERS plays a crucial role in inflammation and obesity.^6^, ^7^ Hence, we have been suggested that ERS could be vital in obesity‐induced AF, and inhibition of ERS might prohibit the development of AF.

## 2 METHODS

### 2.1 Human samples

From September 2018 to March 2019, a total of 14 patients with coronary artery disease and prepared to receive coronary artery bypass graft surgery were enrolled in this study. All patients were excluded from structural heart disease, infectious disease and other severe organ dysfunction. Fourteen patients were divided into the control group (BMI < 25 kg/m^2^) and the overweight group (BMI > 30 kg/m^2^). Right atrium myocardium samples (average 0.1 g) from the incision were collected during the surgery, which would not affect the operation. The samples were immersed into liquid nitrogen immediately and then stored at −80℃ for Western blotting analysis.

The local ethics committee approved the human research. This study was performed in compliance with the Declaration of Helsinki when written informed consents from all recruited patients were acquired.

### 2.2 Animals

C57BL/6J mice provided by Animal Resources of the Chinese Academy of Medical Sciences were adopted as experimental subjects in this study. All mice were randomly assigned into four groups after weaning (21 days) including the control group, the HFD group, the 4‐phenylbutyric acid (4‐PBA) group and the HFD + 4‐PBA group. Mice of the HFD group and the HFD + 4‐PBA group were given high‐fat diet at the age of 4 weeks (60% kcal as fat), provided by the animal feed group of the Experimental Animal Center of Guangdong Province (certificate num: SCXK 2008‐20002), while mice of the control group and the 4‐PBA group were given the normal diet as always. At the age of 20 weeks, the 4‐PBA group and the HFD + 4‐PBA group were given the intraperitoneal injection of 4‐phenylbutyric acid (4‐PBA, 20 mg/kg/day) for consecutive eight weeks, while the control group and the HFD group were given DMSO instead. As previously described, 4‐PBA was first dissolved by DMSO then diluted in 0.9% NaCl.^8^ At the age of 28 weeks, all mice were subjected to the following experiments and then killed to acquire the heart sample. This study followed the recommendations of The Guide for the Care and Use of Laboratory Animals (National Institutes of Health) and was approved by the institutional review boards.

### 2.3 Bodyweight and FBG test

We recorded the bodyweight of four groups of mice every week. At the age of 28 weeks, fasting blood glucose (FBG) of 16 hours was tested with a blood glucose metre.

### 2.4 Intracardiac programmed electrical stimulus

As previously described, we examined the electrophysiological characteristics of all mice via intracardiac programmed electrical stimulus.^9^ Mice were anaesthetized with 2% isoflurane inhalation and fixed on a heated pad at the supine position to maintain 37℃ body temperature. Surface electrocardiogram (ECG) and intracardiac electrogram were recorded simultaneously. P wave duration and an indicator of intra‐atrial conduction time were measured. An electrode catheter (1.1 F, Scisense) was placed into the right atrium through the right jugular vein. A burst of electrical stimuli for 30 seconds was used to test the inducibility of atrial arrhythmias. Three times of burst test were conducted, when more than two times of AF (P wave disappear and last more than 2 seconds) was judged as the onset of AF. LabChart Software 7.0 was used to record and analyse electrocardiogram data.

### 2.5 Western blotting

Proteins were isolated from atrial myocardium samples of patients and mice by M‐PER Mammalian Protein Extraction Reagent (Thermo Fisher Scientific Inc) that contained protease inhibitors and phosphatase inhibitors. Primary anti‐GRP78 (sc‐376768, Santa Cruz), anti‐CHOP (#2895, Cell Signaling Technology), anti‐GAPDH (Bioworld, AP0063), anti‐phospho‐PERK (sc‐32577, Santa Cruz), anti‐PERK (sc‐13073, Santa Cruz), anti‐TGF‐β(Abcam, ab92486), anti‐α‐SMA(Abcam, ab32575), anti‐Collagen I (Abcam, ab34710) and anti‐Collagen III(Abcam, ab7778) were used for Western blotting experiments. Gel Imaging System (Tanon) and Image J software were used to image and analyse Western blotting bands.

### 2.6 Quantitative real‐time PCR

Atrial myocardium samples mice were extracted for total RNA using TRIzol (Takara). 1000 ng total RNA was reversely transcribed into cDNA using the Prime‐Script TMRT reagent kit (Takara). qRT‐PCR was performed using SYBR green (Takara) and normalized to GAPDH expression. Fibroblast‐to‐myofibroblast transition marker of α‐SMA and atrial fibrosis marker of TGF‐β, collagen I, collagen III were examined. Primer pairs for qRT‐PCR in this study were shown in Table 1.

**Table 1:** Primers used for RT‐PCR

Gene	Forward Primer	Reverse Primer
Collagen I	5'‐CATGTTCAGCTTTGTGGACCT‐3’	5'‐GCAGCTGACTTCAGGGATGT‐3’
Collagen III	5'‐TCCCCTGGAATCTGTGAATC‐3’	5'‐TGAGTCGAATTGGGGAGAAT‐3’
TGF‐β	5'‐CCCAGCATCTGCAAAGCTC‐3'	5'‐GTCAATGTACAGCTGCCGCA‐3'
α‐SMA	5'‐CCCACCCAGAGTGGAGAA‐3’	5'‐ACATAGCTGGAGCAGCGTCT‐3'

### 2.7 Masson's trichome staining

Masson's trichrome staining was conducted to evaluate the atrial fibrosis extent of mice. After the sacrifice, the atrium sample of mice was rapidly excised, rinsed with PBS, fixed in 4% paraformaldehyde and embedded in paraffin. Then, five mm‐sections of the atrium were cut. Masson's trichrome kit (#HT15, Sigma Aldrich) was used according to the manufacturer's instruction. Image J was used to evaluate the fibrosis area. Collagen volume fraction (CVF) = Collagen area (blue)/Total area × 100%.

### 2.8 Statistical analysis

We used IBM SPSS Statistics, version 19.0 (SPSS, Inc, Armonk, NY) for statistical analysis. We conducted the normality distribution test of the variables at first to check the variables distribution condition. Continuous variables conformed with normal distribution were presented as mean ± standard deviations, while those not meeting normal distribution were median (lower quartile, higher quartile). Categorical variables were presented as proportions.

For comparison of the overweight group and control group, the Student t test of independent samples was conducted for continuous variables, while Fisher's exact test was performed in different evaluations of categorical variables. For comparison of different groups of mice, one‐way ANOVA and post hoc Tukey's test was achieved when the variables conformed with the normal distribution. Kruskal‐Wallis test and post hoc Dunn's multiple comparisons test were conducted for variables not meeting the normal distribution. A *P*‐value of less than 0.05 was considered statistically significant.

## 3 RESULTS

### 3.1 ERS activation in atrial myocardium of overweight patients

We compared the baseline characteristics and clinical data of overweight patients and control patients. Shown in Table 2, the BMI of the overweight group was much higher than that of the control group (*P* < 0.001). However, there was no significant difference between the two groups in other demographics, echocardiography, laboratory examination, comorbidities and medications (All *P* > 0.05).

**Table 2:** Baseline characteristics and clinical data of control patients and Overweight patients

Variables	Control (n = 8)	Overweight (n = 6)	χ^2^/t	*P*
Demographics				
Age(y)	53.6 ± 7.8	58.1 ± 10.7	0.913	0.379
Gender(%male)	6 (75.0%)	4 (66.7%)	0.117	0.733
BMI (kg/m^2^)	24.5 ± 3.8	30.5 ± 2.5	3.556	<0.001
Smoking	1 (12.5%)	2(33.3%)	0.141	0.707
Echocardiography				
LVEF (%)	56.1 ± 10.4	54.8 ± 6.1	0.295	0.773
LA diameter (mm)	39.8 ± 6.4	44.1 ± 3.9	1.448	0.173
LA volume (mL)	64.8 ± 19.0	73.9 ± 22.4	0.822	0.427
Laboratory examination				
FBG (mmol/L)	5.68 ± 2.15	5.81 ± 1.63	0.123	0.904
HbAlc (%)	5.55 ± 1.32	5.68 ± 1.23	0.188	0.854
hsCRP (mg/L)	1.88 ± 1.15	2.23 ± 1.30	0.533	0.603
BNP (pg/mL)	158.24 ± 78.46	258.41 ± 105.38	2.046	0.063
cTnI (μg/L)	0.06 ± 0.01	0.08 ± 0.03	1.779	0.101
Serum creatinine (μmol/L)	78.52 ± 26.43	73.48 ± 33.12	0.317	0.756
Comorbidities				
Hypertension	3 (37.5%)	3 (50.0%)	0.219	0.640
T2DM	1 (12.5%)	2 (33.3%)	0.141	0.707
AF	0 (0%)	2 (33.3%)	3.111	0.078
COPD	0 (0%)	1(16.7%)	1.436	0.231
Medications				
Aspirin (%)	6 (75.0%)	5 (83.3%)	0.141	0.707
Statins (%)	4 (50.0%)	4 (66.7%)	0.389	0.533
Nitrates (%)	7 (87.5%)	6 (100.0%)	0.807	0.369
ACEI/ARB (%)	2 (35.0%)	1 (16.7%)	0.141	0.707
Amiodarone	0 (0%)	1 (16.7%)	1.436	0.231
Warfarin	0 (0%)	2 (50.0%)	3.111	0.078

Abbreviations: ACEI/ARB, Angiotensin‐converting enzyme inhibitors and angiotensin II receptor blockers; AF, atrial fibrillation; BMI, body mass index; BNP, brain natriuretic peptide; COPD, chronic obstructive pulmonary disease; FBG, fasting blood glucose; HbAlc, Glycated haemoglobin; hsCRP, hypersensitive C‐reactive protein; LA, left atrium; LVEF, left ventricular ejection fraction; T2DM, type 2 diabetes mellitus.

**Figure 1:**
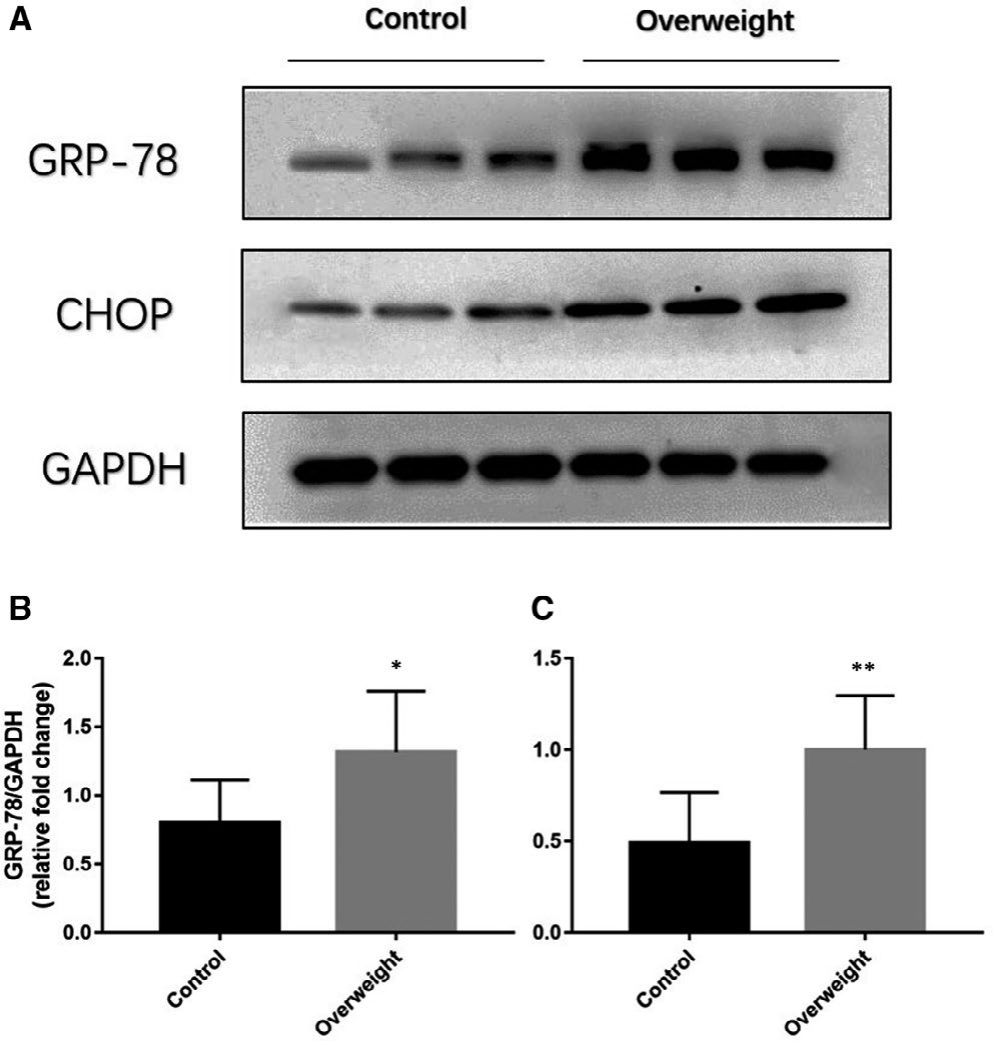
GRP‐78 and CHOP protein expression in atrial myocardium of overweight patients (n = 6) and controls (n = 8). A, Representative Western blotting bands of GRP‐78 and CHOP. B, quantitative analysis of CHOP expression of overweight patients and controls. C, quantitative analysis of CHOP expression of overweight patients and controls. *P < 0.05, **P < 0.01

As previously described,^10^ critical proteins of ERS were examined by WB, which demonstrated that CHOP and GRP‐78 protein expression in the overweight group were significantly higher than those in the control group (both *P* < 0.05), shown in Figure 1. The above data suggested that ERS could be activated in atrial myocardium under the obesity condition.

### 3.2 Bodyweight and FBG

**Figure 2:**
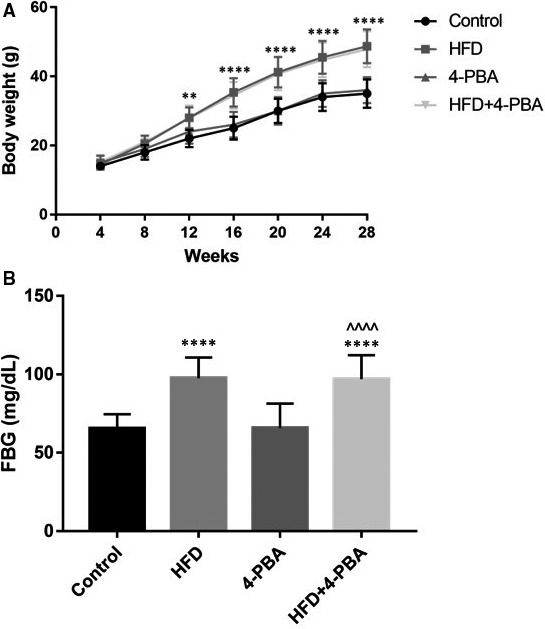
Bodyweight growth curve and FBG level of the control group (n = 15), the HFD group (n = 18), the 4‐PBA group (n = 16) and the HFD + 4‐PBA group (n = 25). A, Bodyweight growth curve of four groups. B, FBG level at 28 wk of four groups. ****P < 0.001 vs Control; ^^^^^^ *P* < 0.001 vs 4‐PBA

Demonstrated in Figure 2A, the bodyweight curves of four groups were recorded, which indicated that the bodyweight of the HFD group and the HFD + 4‐PBA group was significantly higher than that of the control group and the 4‐PBA group at the time point at after 12 weeks. However, the bodyweight was comparable between the HFD group and the HFD + 4‐PBA group, and similar between the control group and the 4‐PBA group. At the age of 28 weeks, we tested the FBG of four groups, which was presented in Figure 2B. FBG level of the control group, the HFD group, the 4‐PBA group and the HFD + 4‐PBA group were (65.4 ± 9.109), (97.5 ± 13.21), (65.8 ± 15.54), (96.9 ± 15.3) mg/dL, respectively. ANOVA results demonstrated that four groups showed a significant difference in FBG level (*F* = 31.36, *P* < 0.001). Post hoc results showed that FBG of the HFD group and the HFD + 4‐PBA group was significantly higher than FBG of the control group and the 4‐PBA group (both *P* < 0.001). However, no disparity was found between the HFD group and the HFD + 4‐PBA group, or the control group and the 4‐PBA group (Both *P* > 0.05).

### 3.3 AF incidence

**Figure 3:**
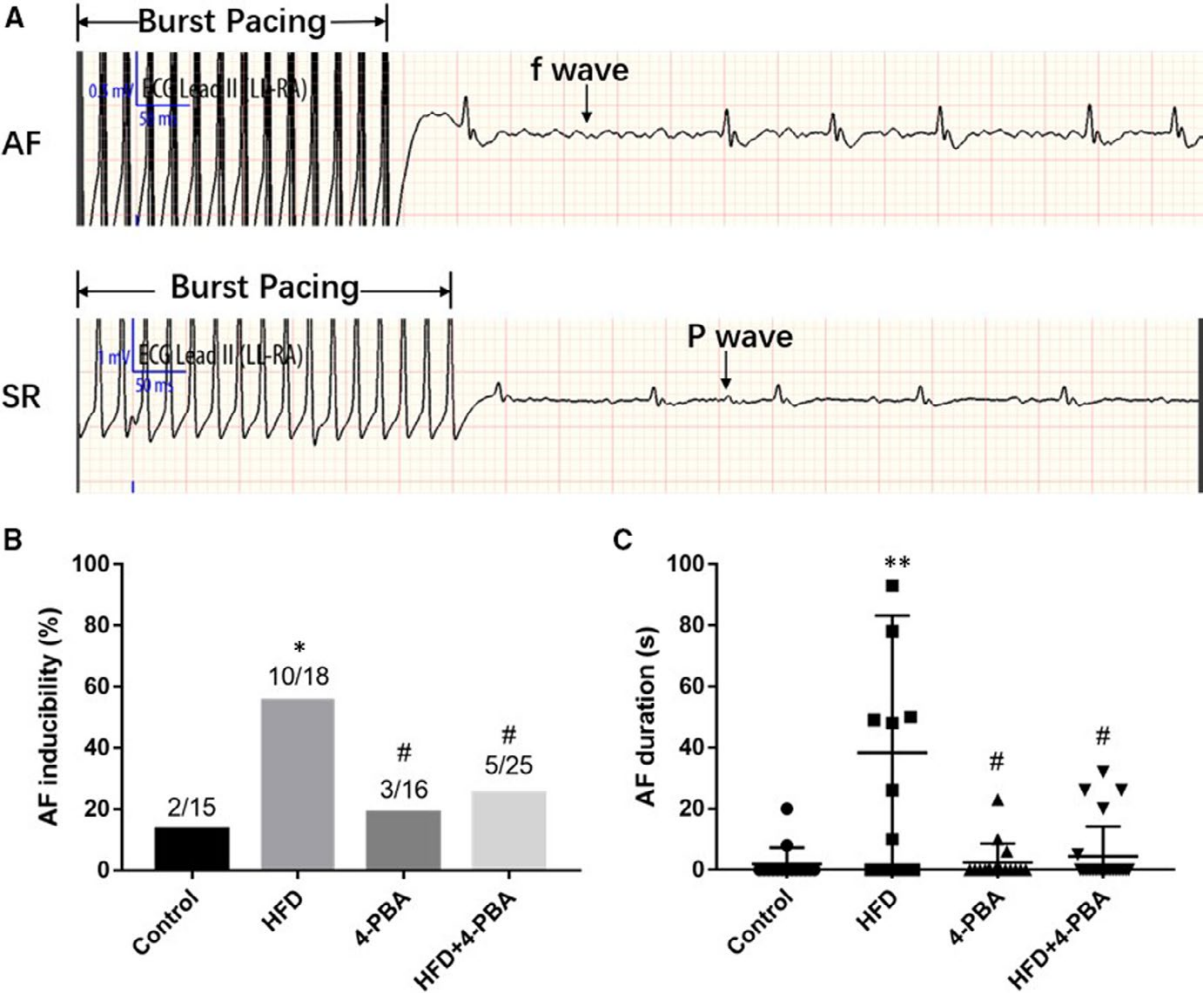
AF incidence and duration examined by the programmed electrical stimulus. A, Recorded representative ECG waves of AF and SR rhythm after a burst of electrical stimuli for 30 s. B, AF incidence of the control group (n = 15), the HFD group (n = 18), the 4‐PBA group (n = 16) and the HFD + 4‐PBA group (n = 25). C, AF duration of four groups. *P < 0.05 vs Control; ^#^ *P* < 0.05 vs HFD

We examined the AF inducibility of four groups, shown in Figure 3. The AF incidence of the control group, the HFD group, the 4‐PBA group and the HFD + 4‐PBA group was 13.3% (2/15), 55.6% (10/18), 18.8% (3/16) and 20.0% (5/25), respectively. The AF incidence of the HFD group was significantly higher than in other groups (*P* = 0.018), while there was no significant difference among the other groups (*P* = 0.862). We also compared the AF duration of all groups, which demonstrated that the AF duration of the HFD group was statistically higher than the other groups (*P* = 0.003).

### 3.4 Atrial fibrosis

**Figure 4:**
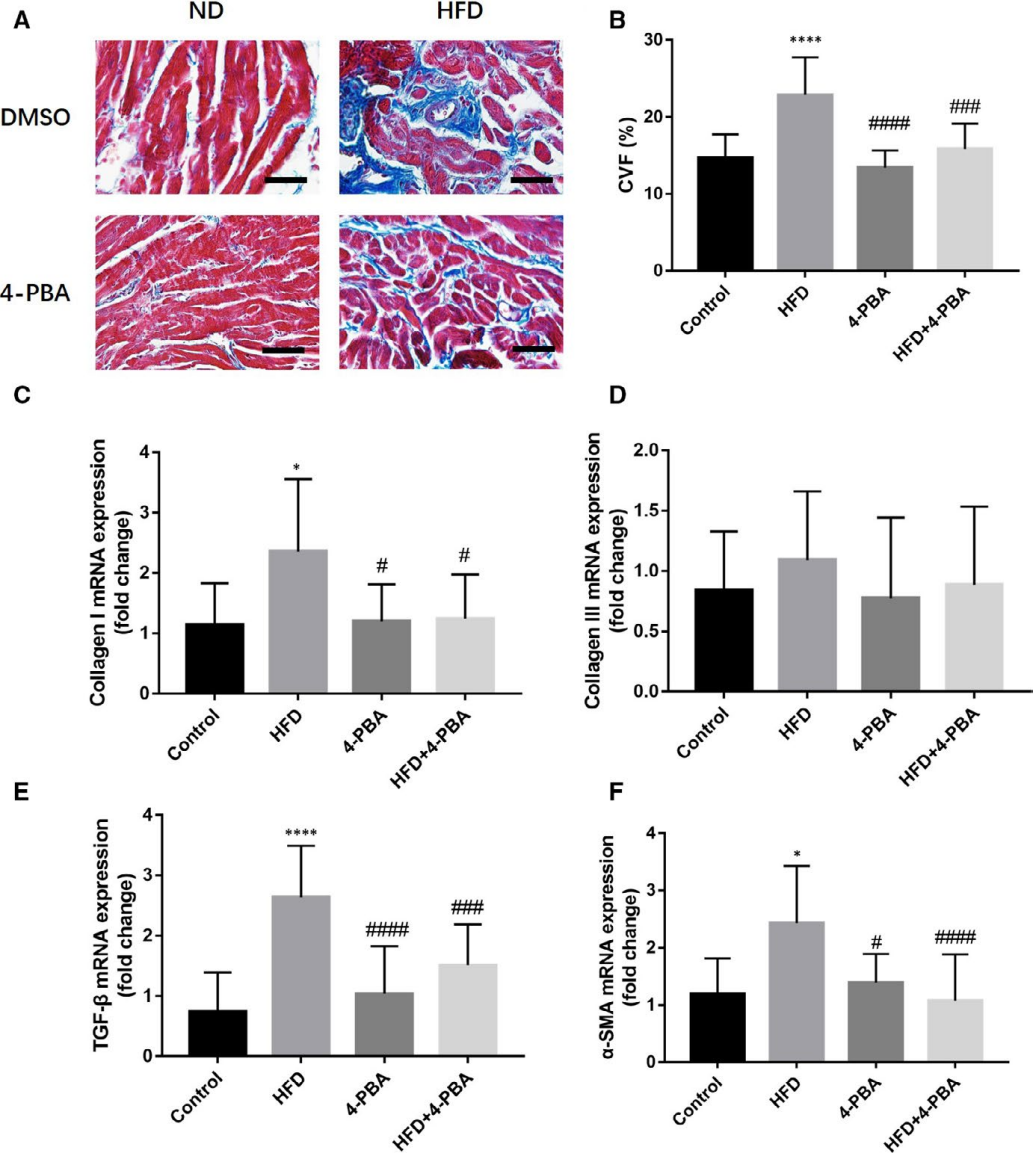
Masson's trichome staining and TGF‐β/α‐SMA/Collagen pathway mRNA examination of the control group (n = 8), the HFD group (n = 8), the 4‐PBA group (n = 8) and the HFD + 4‐PBA group (n = 10). A, Representative sections of four groups, scale bar (200 μm). B, Quantitative analysis of atrial fibrosis. C, Collagen I mRNA expression level. D, Collagen III mRNA expression level. E, TGF‐β mRNA expression levels. F, α‐SMA mRNA expression level. ND, normal diet; HFD, high‐fat diet. CVF, collagen volume fraction. *P < 0.05 vs Control; ****P < 0.001 vs Control; ^#^ *P* < 0.05 vs HFD; ^####^ *P* < 0.001 vs HFD

**Figure 5:**
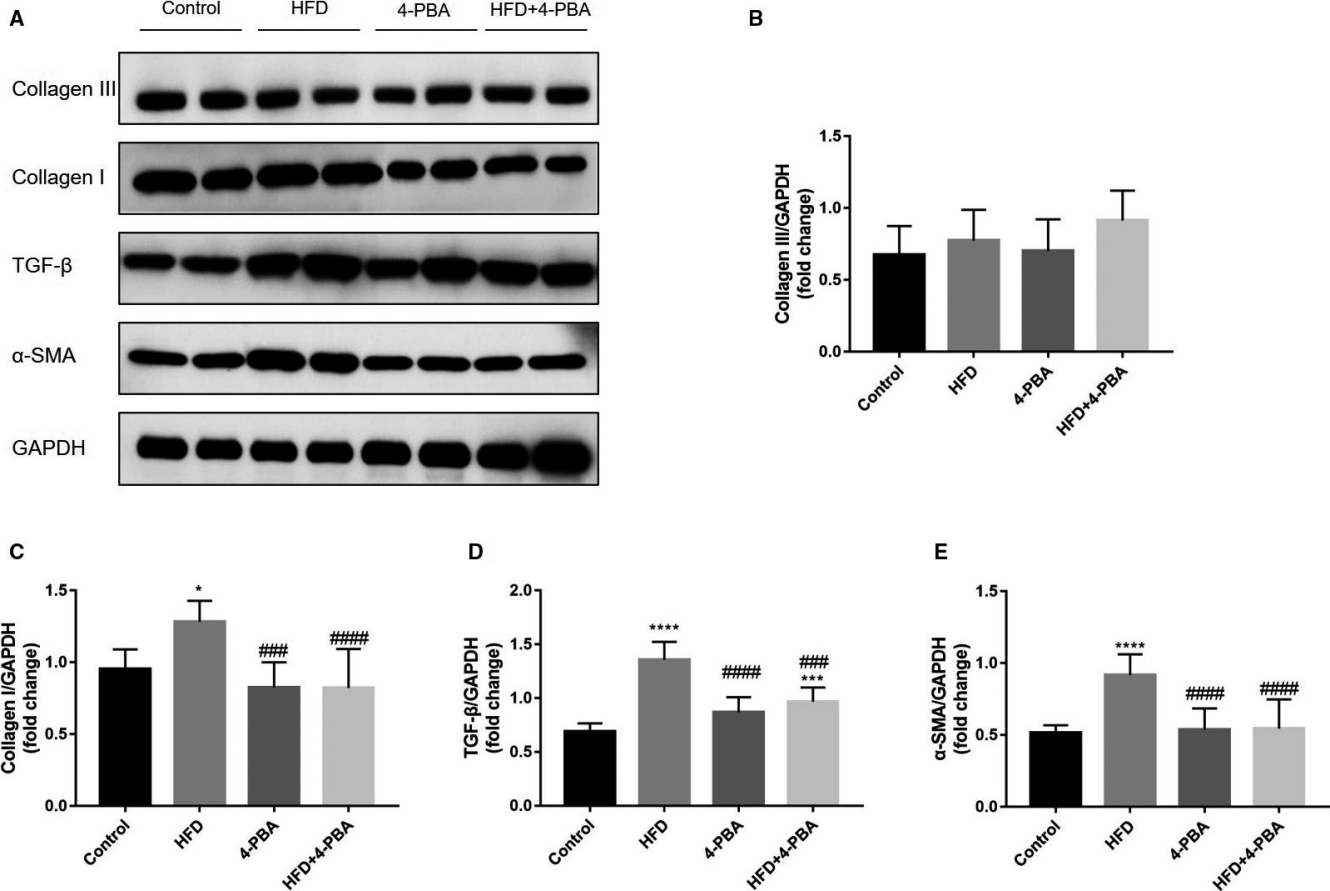
TGF‐β/α‐SMA/Collagen pathway protein expression of the control group (n = 7), the HFD group (n = 10), the 4‐PBA group (n = 8) and the HFD + 4‐PBA group (n = 15). A, Representative Western blotting bands of TGF‐β, α‐SMA, Collagen I and Collagen III. B, Quantitative analysis of Collagen III expression. C, Quantitative analysis of Collagen I expression. D, Quantitative analysis of TGF‐β expression. E, Quantitative analysis of α‐SMA expression. *P < 0.05 vs Control; ***P < 0.005 vs Control; ****P < 0.001 vs Control; ^###^ *P* < 0.005 vs HFD; ^####^ *P* < 0.005 vs HFD

Masson trichome's trichome staining was conducted to evaluate the atrial fibrosis of all mice. Representative sections and mRNA expression levels of critical markers were expressed in Figure 4. HFD mice had significantly more severe atrial fibrosis than the control mice (*P* < 0.001), while CVF was similar between the 4‐PBA group and the control group (*P* = 0.879). However, the CVF of HFD + 4‐PBA was significantly lower than that of the HFD group (*P* = 0.001). We used qRT‐PCR to test the TGF‐β/α‐SMA/Collagen pathway, which demonstrated that it was activated in HFD mice. Compared with the HFD group, the TGF‐β/α‐SMA/Collagen pathway markers of HFD + 4‐PBA were significantly decreased. To further validate the TGF‐β/α‐SMA/Collagen pathway expression, we evaluated the protein expressions, shown in Figure 5. Consistent with qRT‐PCR results, the TGF‐β, α‐SMA and Collagen I protein expressions was significantly increased in the HFD group, which was also higher than the 4‐PBA group and the HFD + 4‐PBA group.

### 3.5 ERS activation in atrial myocardium of mice

**Figure 6:**
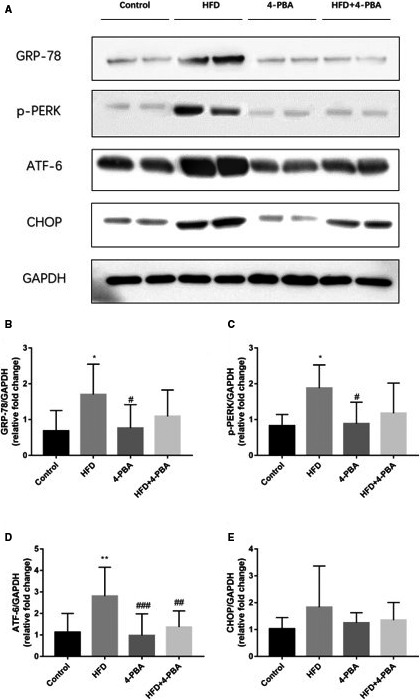
ERS key markers protein expression of the control group (n = 7), the HFD group (n = 10), the 4‐PBA group (n = 8) and the HFD + 4‐PBA group (n = 15). A, Representative Western blotting bands of GRP‐78, p‐PERK, ATF‐6, CHOP. B, Quantitative analysis of GRP‐78 expression. C, Quantitative analysis of p‐PERK expression. D, Quantitative analysis of ATF‐6 expression. E, Quantitative analysis of CHOP expression. *P < 0.05 vs Control; **P < 0.01 vs Control; ^#^ *P* < 0.05 vs HFD; ^##^ *P* < 0.01 vs HFD; ^###^ *P* < 0.005 vs HFD

The key markers of ERS activation, including GRP‐78, p‐PERK, ATF‐6 and CHOP protein levels of four groups, were also examined, shown in Figure 6. We found that all of those proteins were highly expressed in atrial myocardium of HFD mice. However, those proteins of the HFD + 4‐PBA group were significantly down‐regulated than the HFD group. Also, no statistical difference was observed between the control group and the 4‐PBA group in protein expression levels.

## 4 DISCUSSION

With the economic development and changes in the disease spectrum, obesity is gradually becoming a major global threat to human health, which dramatically increases the risk of death and cardiovascular disease.^11^ Atrial fibrillation, the most common sustained arrhythmia in clinics, is strongly associated with obesity, with increased risk of higher incidence, prevalence, severity, and progression led by obesity.^3^ In this study, we first build the obesity mice model by giving HFD, then validate the AF incidence of obesity mice. What's more, we evaluated the ERS activity of different groups and the efficacy of ERS inhibition in preventing AF. The followings are we have found: 1. Clinical findings demonstrated that ERS key marker expression was elevated in atrium tissue of overweight patients, and a similar situation happens in HFD mice; 2. HFD mice were subjected to higher body weight and elevated FBG, but not high enough for type 2 diabetes mellitus (T2DM); 3. HFD mice had higher AF incidence and duration, which could be attributed to more severe atrial fibrosis; 4. Inhibition of ERS with 4‐PBA could mitigate not only the atrial fibrosis but also limit the AF incidence and duration.

ERS, oxidative stress and inflammatory responses are closely interacted and compose a network helping the cells adapt to and survive stress conditions caused by all kinds of biochemical, physiological and pathological stimuli.^12^ Obesity is a chronic pathological stimulus associated with insulin resistance, characterized by a state of low‐grade inflammation manifested in elevated levels of proinflammatory cytokines in blood and tissues.^13^ Previous studies have demonstrated the vital role of ERS in the pathogenesis and comorbidities of obesity. Contreras and his colleagues found that rats fed a high‐fat diet displayed hypothalamic ER stress, while modulation of hypothalamic GRP78 activity is associated with browning in WAT.^14^ Another study demonstrated that targeting ERS could ameliorate retinal inflammation caused by HFD.^15^ High‐fat diet and streptozotocin‐induced diabetes could lead to the renal lesion, and endoplasmic reticulum stress and CHOP may contribute to the injury process.^16^ Despite numerous clues of ERS in HFD‐induced inflammation and stress, there was minimal evidence of the role of ERS in AF. To assess the role of ERS in obesity and AF, we tested the ERS activity in overweight patients and controls, which demonstrated that the ERS process was activated in atrial myocardium of obese patients, in line with previous reports.^17^ We built the obesity mouse model by giving HFD, and their bodyweight and FBG surpassed controls. Notably, there was no difference between HFD group and HFD + 4‐PBA group in bodyweight and FBG, indicating that 4‐PBA and ERS have no significant impact on glucose metabolism and body weight. We also validated the clinical findings by exploring ERS pathways in atrial myocardium of mice, demonstrating that ERS markers, including p‐PERK, GRP‐78, CHOP and ATF‐6, were all elevated in atrium tissue of HFD group. These results proved that ERS was activated and increased in the atrium of obese people and mice.

Obesity dramatically increases the risk of AF in multiple ways. Fukui and his colleagues found that HFD mediated AF incidence and atrial fibrosis, which is associated with hyperleptinemia. When the leptin gene was knocked down (leptin‐deficient ob/ob mice), the AF incidence and atrial fibrosis would be significantly mitigated.^18^ We also found that the HFD group had significant atrial fibrosis compared with the control group. However, another study led by Maria Z found that HFD‐fed mice developed obesity, insulin resistance and vulnerability to AF, but no atrial fibrosis.^19^ Although Maria reported no atrial fibrosis, her study also demonstrated that some critical markers of atrial fibrosis were significantly up‐regulated, including TGF‐β and MMP‐9, indicating the start of fibrotic process and an pre‐fibrosis condition in this model. This difference could be attributed the different model building time. Maria Z examined the atrium at the age of 24 weeks, while we conducted the experiments at 28 weeks. We examined the mRNA and protein expression levels of the TGF‐β/α‐SMA/Collagen pathway in different groups, which was significantly higher in the HFD group, indicating that cardiac fibroblast was activated and produced Collagen. Notably, we found that only Collagen I was increased in the HFD group, while Collagen III was similar among all groups. Hua Shen also found that TGF‐beta1/alpha‐SMA/Col I profibrotic pathway was activated in AF rabbit pacing models.^20^

Some studies demonstrated that IL‐10,^21^ connexin 40 (C×40) and C×43^22^ could participate in the atrial electrical remodelling^23^, ^24^ and structural remodelling induced by HFD. Our study demonstrated that ERS was activated in HFD mice, while HFD mice have significant atrial fibrosis and higher incidence of AF. To figure out the cause result relationship, we applied the inhibitor of ERS, 4‐PBA, which is a widely used inhibitor of ERS due to its specificity.^25^ Hong applied 4‐PBA in preventing vital organ injury in acute rat pancreatitis and found that ERS was an essential player in the development of severe pancreatitis‐induced multiorgan damage.^26^ Another study demonstrated that 4‐PBA could selectively attenuate ER stress and might be a potential therapeutic target in protecting the heart from pressure‐overload induced myocardial hypertrophy and interstitial fibrosis.^8^ This present study also proved that 4‐PBA could significantly reduce atrial fibrosis and the incidence of AF induced by HFD, which also validated the crucial role of ERS in the process of AF caused by obesity. Despite the effectiveness of 4‐PBA on ERS, it still requires further validation and examination of side effects before pre‐clinical application. However, it was reported that a patient with benign recurrent intrahepatic cholestasis type 2 was administered with 4‐PBA successfully without apparent side effects.^27^

Several limitations of this study must be noted. Firstly, more clinical pieces of evidence were required to confirm the roles of ERS in AF and obesity, including more samples and more in‐depth investigations. Secondly, transgenic mice might be more persuasive in demonstrating this hypothesis, which could be applied in further studies. Last, the underlying mechanism requires more exploration, including the specific signalling pathway.

## 5 CONCLUSIONS

In summary, obesity is associated with ERS activation in the atrium, which could be a key player in the pathogenesis of atrial fibrosis and the development of AF. Inhibition of ERS via application of 4‐PBA could mitigate the ERS activity, mitigate the atrial fibrosis process, and reduce the incidence and duration of AF induced by HFD.

## CONFLICT OF INTEREST

The authors declare no conflicts of interest.

## AUTHOR CONTRIBUTION

**Yan Zhang:** Conceptualization (equal); Data curation (equal); Writing‐original draft (equal). **Shuwen Yang:** Formal analysis (equal); Investigation (equal); Methodology (equal). **Jing Fu:** Data curation (equal); Formal analysis (equal); Methodology (equal); Resources (equal). **Annan Liu:** Resources (equal); Validation (equal). **Deping Liu:** Investigation (equal); Methodology (equal); Visualization (equal). **Suyan Cao:** Conceptualization (equal); Funding acquisition (equal); Resources (equal); Writing‐review & editing (equal).

## Data Availability

The data that support the findings of this study are available from the corresponding author upon reasonable request.
